# The *Francisella *pathogenicity island protein IglA localizes to the bacterial cytoplasm and is needed for intracellular growth

**DOI:** 10.1186/1471-2180-7-1

**Published:** 2007-01-17

**Authors:** Olle M de Bruin, Jagjit S Ludu, Francis E Nano

**Affiliations:** 1 The Department of Biochemistry and Microbiology, University of Victoria, Victoria, B.C., Canada

## Abstract

**Background:**

*Francisella tularensis *is a gram negative, facultative intracellular bacterium that is the etiological agent of tularemia. *F. novicida *is closely related to *F. tularensis *but has low virulence for humans while being highly virulent in mice. IglA is a 21 kDa protein encoded by a gene that is part of an *iglABCD *operon located on the *Francisella *pathogenicity island (FPI).

**Results:**

Bioinformatics analysis of the FPI suggests that IglA and IglB are components of a newly described type VI secretion system. In this study, we showed that IglA regulation is controlled by the global regulators MglA and MglB. During intracellular growth IglA production reaches a maximum at about 10 hours post infection. Biochemical fractionation showed that IglA is a soluble cytoplasmic protein and immunoprecipitation experiments demonstrate that it interacts with the downstream-encoded IglB. When the *iglB *gene was disrupted IglA could not be detected in cell extracts of *F. novicida*, although IglC could be detected. We further demonstrated that IglA is needed for intracellular growth of *F. novicida*. A non-polar *iglA *deletion mutant was defective for growth in mouse macrophage-like cells, and *in cis *complementation largely restored the wild type macrophage growth phenotype.

**Conclusion:**

The results of this study demonstrate that IglA and IglB are interacting cytoplasmic proteins that are required for intramacrophage growth. The significance of the interaction may be to secrete effector molecules that affect host cell processes.

## Background

*Francisella tularensis *is the etiological agent of the severe, febrile disease tularemia. Although there have been rare isolates of *F. tularensis *in Australia, tularemia is mainly a disease of the Northern hemisphere that is spread by blood-sucking mosquitoes, flies, and ticks or acquired from contact with infected animals such as rabbits, rodents, and beavers [1]. Occasionally, local outbreaks of tularemia are associated with contact or consumption of contaminated natural water. In addition, *F. tularensis *is potentially a threat as a bioterrorist agent due to its high infectivity and lethality when inhaled. *F. novicida *is highly related at the DNA level to *F. tularensis*, and serves as a model organism since it is very virulent in mice while being avirulent in humans.

*F. tularensis *is a gram-negative, facultative intracellular bacterium capable of survival and replication in macrophages [2]. A common virulence strategy of intracellular pathogens is to favorably modulate the intracellular milieu of hosts for their own benefit. In *Legionella pneumophila *a type IV secretion system (T4SS) delivers effectors that allow the pathogen to replicate in ribosome-studded phagosomes that fail to fuse with lysosomes [3,4]. *Salmonella enterica *relies on a pathogenicity island-encoded type III secretion system (TTSS) to modify phagosome biogenesis [5,6], including inhibition of phago-lysosomal fusion [7] and the NADPH oxidase-mediated killing by host cells [5]. Other intracellular pathogens, such as *Listeria monocytogenes*, degrade the phagosomal membrane and escape into the cytoplasm to replicate freely [8]. *F. tularensis *initially resides in a phagosome which accumulates some late endosome markers. After about four hours most *F. tularensis *cells escape the phagosome and grow in the cytoplasm. [2,9-11]. Although an intact *iglC *gene is needed for *F. tularensis *to escape phagosomes, the role of IglC is unknown.

We recently described a *Francisella *pathogenicity island (FPI) harboring several genes necessary for intracellular growth. Four FPI genes, *iglABCD*, are organized in an apparent operon [12]. The production of IglC mRNA is in part dependent on MglA [13] which is thought to be a global regulator of virulence factors in *F. tularensis*. By analogy with its *Escherichia coli *homologue, SspA, MglA likely interacts with RNA polymerase to directly or indirectly alter transcription of several genes [14]. Disruption of *mglA *or *mglB *results in mutants that are severely attenuated for virulence [15]. IglC has been shown to be induced about four-fold during intracellular growth relative to broth growth and necessary for virulence [16-18], and it was recently demonstrated that inactivation of *iglC *and *mglA *result in mutants that remain in phagosomes that fuse with lysosomes [19,20]. Although an *iglA *transposon insertion mutant has been shown to be defective for intracellular growth, it could not be ruled out that the observed phenotype was due to interruption of transcription of downstream genes, including *iglC *[17].

In this study, we use *F. novicida *to investigate the properties of IglA and its role in *F. novicida *intracellular growth. *F. novicida *is particularly suited for these studies since, unlike *F. tularensis*, it contains only one copy of the FPI, and this simplifies the construction of mutants. Further, the biology of *F. novicida *growth in human macrophages is indistinguishable from that of *F. tularensis *strains [9,11], and thus *F. novicida *serves as a valid surrogate for virulent strains when studying basic aspects of *Francisella *intracellular growth. In this work we supply evidence that IglA is a cytoplasmic protein that interacts with IglB, and is required for intramacrophage growth.

## Results

### IglAB homologues in diverse bacteria are organized in a conserved gene cluster

Homologues of *iglA *and *iglB *exist in several bacterial species that are either animal or plant pathogens or plant symbionts [12] but there are no known homologues of *iglC *or *iglD*. IglAB homologues in *Vibrio cholerae, Salmonella enterica, Rhizobium leguminosarum*, and other bacteria are found in a cluster of genes encoding proteins known as IcmF-associated homologous proteins (IAHPs) [21-23]. Recently, it was demonstrated that this gene cluster encodes components of a proposed type VI secretion system (T6SS) in *Vibrio cholerae *[24].

**Figure 1:**
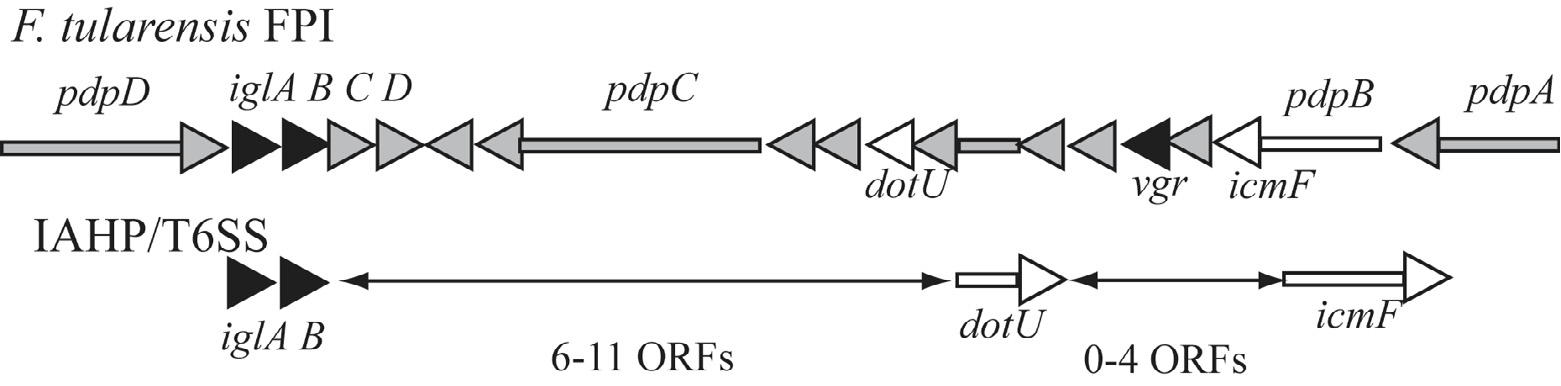
**Similarity of the FPI to other virulence gene clusters**. Homologues of *Francisella *pathogenicity island proteins IglA and IglB are found on a conserved gene clusters known as IcmF associated homologous proteins (IAHP), which, in some cases encode a proposed type VI secretion system. In the FPI the IcmF motif appears at the C-terminus of PdpB. Downstream of the *pdpB *gene is an ORF designated "*vgr*" that encodes a protein with similarity the Vgr family, one of which is secreted by the proteins encoded by an IAHP cluster in *Vibrio cholerae*. Homologues of the *Legionella dotU *gene are often associated with IAHP clusters. A very weak similarity to *dotU *is seen in an ORF that is sixth downstream of *pdpB*.

In light of the emerging role of IAHP/T6SS in the secretion of proteins we re-examined the ORFs in the FPI to determine if components of a type VI secretion system may be present. Three essential components of a T6SS are a protein with an IcmF-motif and two linked genes that correspond to *iglA *and *iglB*. A BLASTP search revealed that an IcmF region was found as part of the C-terminal third of PdpB which aligned with the corresponding regions of proteins belonging to the IcmF conserved orthologous group (COG3523.2 with an E-value of 7 × 10^-9^). The identification of IglA and IglB as members of COGs is much clearer. IglA has strong identity to members of COG3516 (E-value of 2 × 10^-20^) and IglB has strong identity with COG3517 (E-value of 2 × 10^-102^). Remarkably all of the relatives of *iglAB *are organized in the same order, and are always adjacent to each other on the chromosome. The *iglAB *genes together with an *icmF*-containing gene form the core set of genes that suggest the presence of a type VI secretion system. We also found through BLASTP analysis that the deduced product of an ORF 380 bp downstream of *pdpB *(shown as "*vgr*" in Fig. 1) shows a weak similarity (E-value 0.15) to the family of *vgr*-encoded proteins, such as VgrG [24] which is secreted by a T6SS in *V. cholerae*. Vgr proteins are hydrophilic proteins that contain valine-glycine repeats, and are found in a number of gram negative pathogens. Another ORF, 4587 bp downstream of *pdpB *show similarity (E-value, 0.0005) to proteins in COG3455 that includes the IAHP-associated protein DotU. The clustering of *iglAB *and the *icmF*-containing *pdpB *gene, together with two other IAHP-associated genes strongly suggests that the FPI carries a type VI secretion system.

### IglA expression in an *mglAB *background

**Figure 2:**
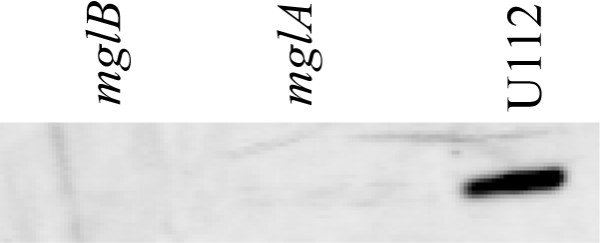
**IglA regulation by MglA and MglB**. Western blot showing lack of IglA in *mglA *and *mglB *mutants but present in the wild type strain U112. All samples were normalized to 6 μg protein per lane.

Previously RT-PCR analysis of the level of *iglA*, *iglC *and *iglD *transcripts revealed a role of MglA in regulating expression of the *iglABCD *operon mRNA production [13]. We wished to test if IglA protein expression levels are depressed in mutant *mglA *and *mglB *backgrounds. Western immunoblot analysis of IglA in an *mglA *mutant and an *mglB *background revealed that IglA is not expressed at detectable levels in these strains (Fig. 2).

### IglA expression during intramacrophage growth

**Figure 3:**
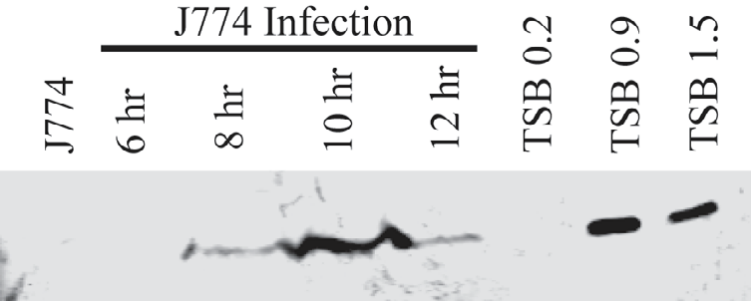
**IglA expression in J774 macrophages**. Western blot showing expression of IglA during infection of macrophages. J774 macrophages were infected with parent strain U112 (m.o.i 300:1) and lysed at the indicated time post infection. Loading was normalized according to the number of viable bacteria (CFU) in each sample as determined by plating on TSA-C plates. Lane J774, uninfected macrophages. TSB, broth grown U112 grown to indicated optical density (600 nm). All samples were normalized to 10^7 ^CFU by viable counts. The macrophage cell lysates altered the appearance of the IglA bands, but control experiments showed that the cell lysates did not mask IglA reactivity with antibody.

Previous studies provide evidence that MglA expression peaks at about 5 hours after infection of macrophages [25], and that IglC expression is maximal at between 6 and 24 hours after infection [16]. To access the pattern of IglA expression during *F. novicida *infection of macrophages, we lysed J774 macrophages at various time points after infection with the wild type strain U112 and examined the lysates for IglA using immunoblotting. In our assays IglA was first detectable at 8 hours post-infection, peaked at 10 hours, and showed a decline by 12 hours (Fig. 3). In broth grown cultures IglA appeared to be maximally expressed at the late logarithmic phase of growth.

### IglA is cytoplasmically located

**Figure 4:**
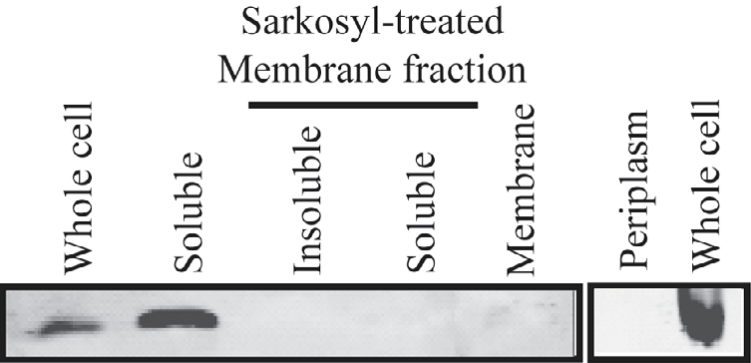
**Subcellular localization of IglA**. Anti-IglA was used to probe Western immunoblot of subcellular fractions of *F. novicida*. The sarkosyl insoluble fraction represents an enrichment of outer membrane protein and the sarkosyl soluble fraction contains largely inner membrane protein. Samples were prepared as outlined in Methods and normalized to 10 μg protein per lane before separation on a 12% SDS-PAGE gel. Results are representative of three independent experiments.

Knowing the cellular localization of a protein can help lead to a hypothesis as to its biological role. To investigate the subcellular localization of IglA, we fractioned *F. novicida *U112 into soluble and membrane-associated fractions and determined the amount of IglA in each fraction by immunoblot analysis. The data from this experiment revealed that IglA is exclusively a soluble protein (Fig 4). Although IglA lacks a signal peptide sequence, it could not be ruled out that IglA localizes to the periplasm by a novel mechanism. Therefore, we isolated the periplasmic contents from *F. novicida *and determined by immunoblotting that IglA does not localize to this compartment. We also failed to detect IglA in culture supernatant (data not shown). The data from these experiments strongly suggest that IglA is a cytoplasmic protein. In agreement with this, the IglA homologue in *Salmonella enterica *has been predicted to be localized to the cytoplasm [23].

### IglA interacts with IglB in vivo

**Figure 5:**
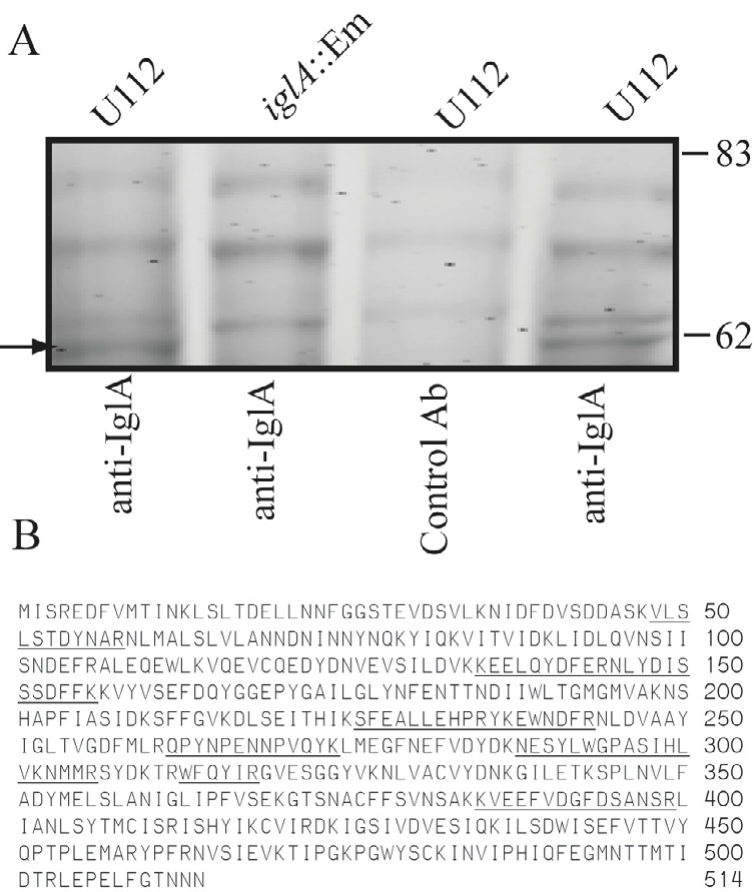
**Co-immunoprecipitation of a 60 kDa protein with IglA**. Panel A. Anti-IglA serum co-immunoprecipitates a circa 60 kDa soluble protein (arrow, lanes 1 and 4). The band is absent in control reactions with non-specific antibody (lane 3) and in immunoprecipitations with an *iglA *mutant (lane 2). Numbers shown indicate molecular mass standards. Results are representative of those of three experiments. Panel B. MALDI-TOF identified the 60 kDa protein as IglB. Underlined sequences indicate peptides identified by MALDI-TOF. The second and third regions each represent two peptides (break after the "R"). Of 25 queries submitted, 9 showed significant identity with rabbit heavy chain and 9 showed significant identity with IglB of *F. novicida*. No other significant hits were found in the MSDB 20060224 databank.

To investigate interactions of IglA with other *F. novicida *proteins we performed immunoprecipitations with anti-IglA antibody on soluble proteins. A co-precipitating protein with a relative molecular mass of approximately 60 was detected (Fig 5A). This protein band was excised and subjected to MALDI-TOF analysis, and the resulting peptide fragment masses were submitted to searches against predicted peptide fragments of prokaryotes in the MASCOT data bank. This analysis revealed that the only significant match was IglB from *F. novicida *(Fig. 5B). The relative molecular mass of the co-precipitated protein is consistent with this result as IglB is predicted to be 58 kDa. Immunoprecipitations performed with an *iglA *null strain did not result in the appearance of the 60 kDa band, nor did immunoprecipitations of U112 done with pre-immune serum. These results strongly suggest that IglA and IglB interact in the cytoplasm of *F. novicida*.

Supporting the hypothesis that IglA interacts with IglB is the finding that IglB mutants but not IglC mutants lack detectable IglA (see below, Fig. 8). Presumably a lack of association of IglA with IglB makes the former susceptible to degradation.

### Deletion mutagenesis of *iglA *and complementation of the mutant strain

**Figure 6:**
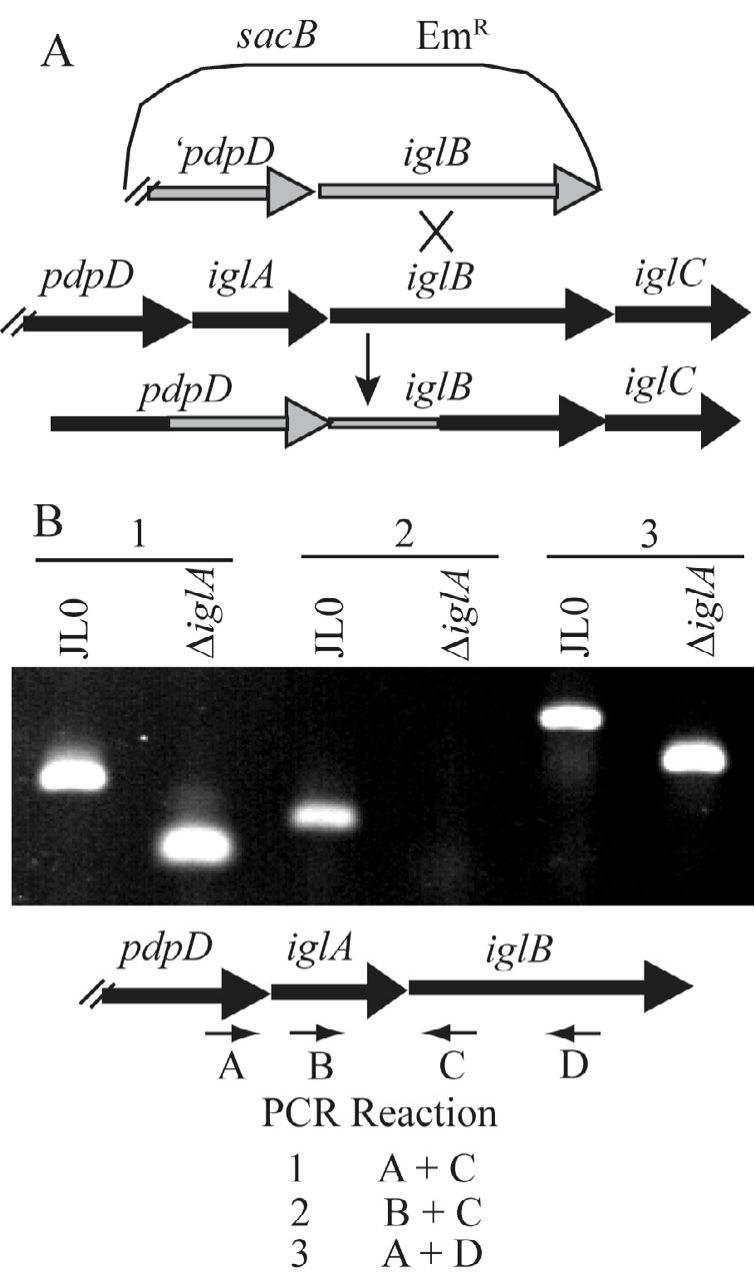
**Deletion mutagenesis of *iglA***. Panel A. Diagram of steps used to construct an *iglA *deletion mutant. A fragment of *pdpD *was joined to *iglB *and these two fragments were ligated to an Em^R^-*sacB *cassette. After transformation the recombinant construct integrated into the *F. novicida *chromosome. Plating the strain with the integrated fragment on sucrose selected for strains that had undergone an excision of the *sacB *and neighboring regions. Panel B. PCR confirmation of the deletion of *iglA*. The small arrows indicate the location of the primers used in the reactions.

**Figure 7:**
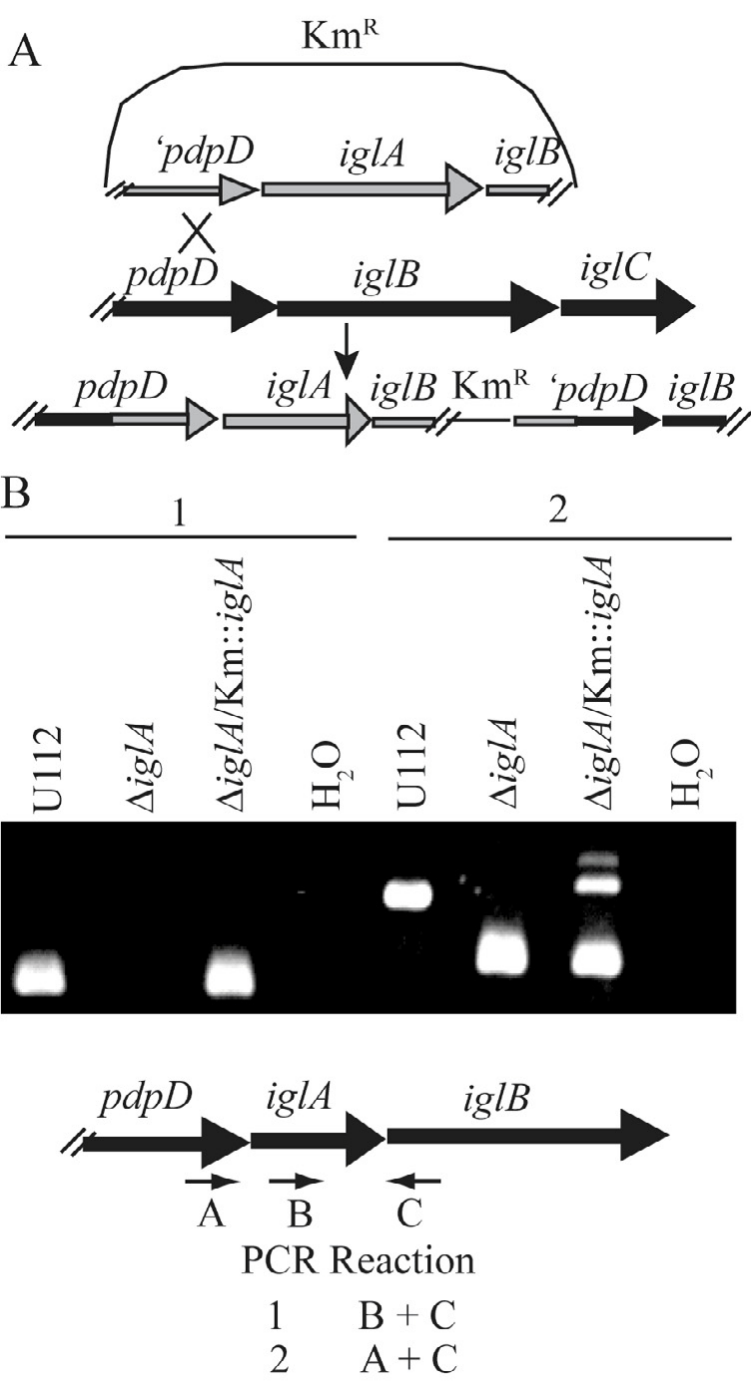
**In cis complementation of *iglA***. Panel A. Diagram of complementation scheme. A PCR amplicon containing the *iglA *and neighboring regions was ligated to a Km^R ^cassette and used to transform a Δ*iglA *strain. Integration of the recombinant construct resulted in a strain with a chromosomally-integrated *iglA*. Panel B. PCR reactions demonstrating the presence of *iglA *in the complemented strain. Arrows in lower part of diagram indicate the location of the PCR primers used in the reactions.

**Figure 8:**
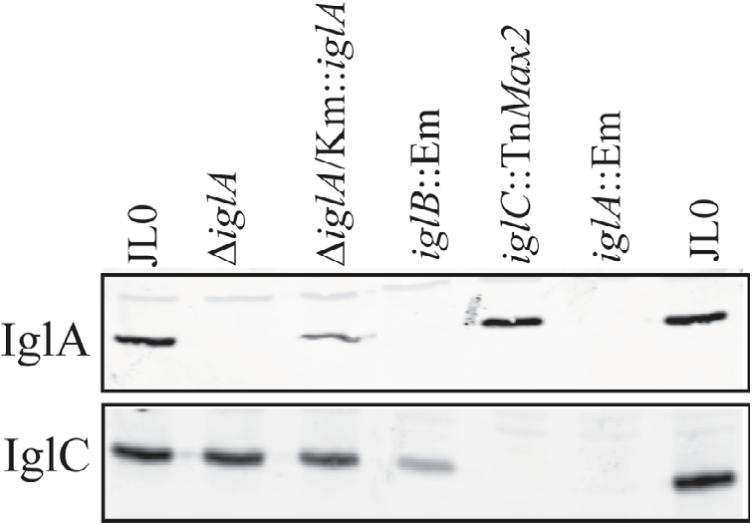
**An *iglA *mutant lacks the expression of a 21 kDa protein**. Western blot showing the lack of an anti-IglA serum reactive 21 kDa protein in the Δ*iglA *strain (top panel). Wild type levels of IglC are retained in the Δ*iglA *strain (bottom panel). In contrast, the *iglA*::Em mutant lacks expression of IglC. The expression of IglC is threefold lower in an *iglB*::Em strain than in JLO and Δ*iglA*. Fluorescence intensity was used to quantify relative amounts of protein.

An *iglA *deletion mutant, ODB2, was constructed using a two-step integration-excision method (Fig. 6A). First, the PCR-amplified 1.5 kbp regions flanking *iglA *were joined so as to leave *iglB *intact, including its ribosome binding region. This recombinant construct was ligated to an erythromycin resistance-*sacB *cassette and the ligation mixture was used to chemically transform *F. novicida *JL0 to erythromycin resistance. The JL0 strain is a derivative of U112 that has a deletion in one of its putative sucrose hydrolase genes, and is thus sensitive to sucrose when *sacB *is expressed. This strain behaves like wild type in our virulence assays (data not shown). An erythromycin resistant colony was grown and plated on agar media containing 10% sucrose which acts as a counter selective marker for the *sacB *gene. Sucrose sensitive strains were examined for loss of *iglA *by PCR (Fig. 6B). Attempts to genetically complement the Δ*iglA *strain by incorporating *iglA *into a *F. tularensis *plasmid pFNLT1 [26] failed, presumably because the over-expression of IglA was lethal to *F. novicida*. Hence, an *in cis *complementation approach was devised, allowing *iglA *to be incorporated into the chromosome linked to a kanamycin resistance marker (Fig. 7A and 7B). The *iglA *deletion strain failed to produce IglA as determined by Western immunoblotting (Fig. 8). However, the Δ*iglA *strain retained expression of IglC at parental strain levels. *In cis *complementation of the Δ*iglA *strain resulted in a strain that regained partial expression of IglA. An insertion mutant of *iglB *gave a reduction in the amount of IglC that was made, and this is not surprising since many insertion mutation decrease the expression of downstream genes. Surprisingly, this same mutant lacked expression of IglA, suggesting that the co-expression of IglB is needed for expression of IglA or to prevent degradation of IglA. Disruption of *iglC *however, does not affect the amount of IglA detected (Fig. 8).

### IglA is required for growth in the J774 macrophage cell line

**Figure 9:**
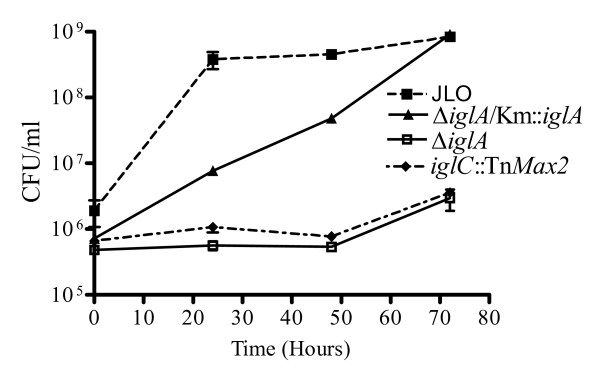
**IglA is required for intracellular growth**. Growth of Δ*iglA *strain in J774 mouse macrophage-like cells. Filled squares, parental strain JLO; open squares, Δ*iglA*; triangles, *in cis *complementation strain; diamonds, *iglC *transposon insertion mutant CG62. The experiments were done in triplicate and standard errors are shown by bars. This graph shows data from one of three independent experiments.

Previous work has suggested that IglA is required for *F. novicida *intramacrophage growth and virulence; however, its role has never been unequivocally demonstrated. In order to assess the requirement for IglA expression in intramacrophage growth we used our defined deletion and complemented strains to infect a culture of the J774 macrophage cell line. The data shown in figure 9 illustrates that the Δ*iglA *strain is incapable of intramacrophage growth, as is the *iglC *negative strain, CG62. The Δ*iglA *strain that was complemented for IglA production partially regained its ability to grow in macrophages. The residual defect in intracellular growth is not unexpected since we showed that the expression of IglA was not at wild type levels. Δ*iglA *replicated as the parental strain in broth (data not shown).

### The ΔiglA strain has lowered virulence in chicken embryos

**Figure 10:**
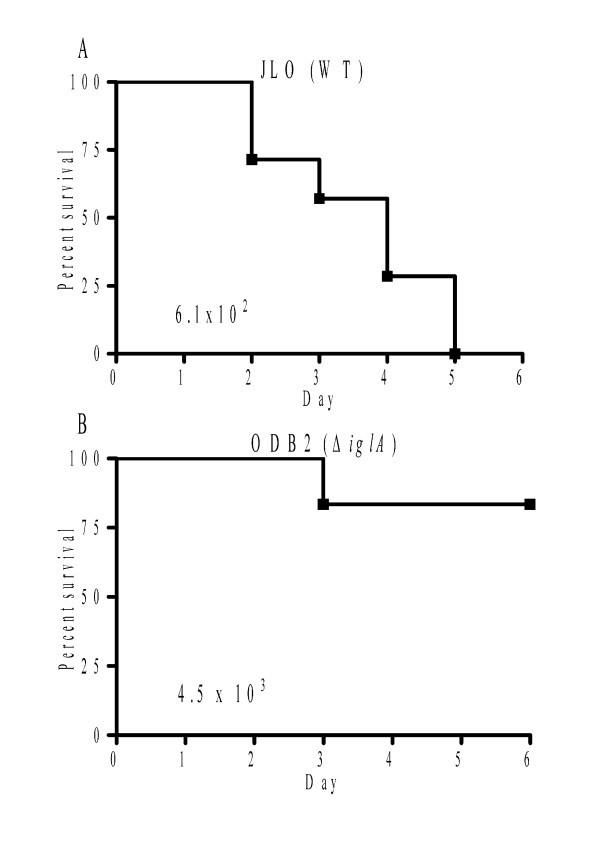
**Δ*iglA *mutant is less virulent in chicken embryos**. Infection of chicken embryos with 600 CFU of wild type (JL0) *F. novicida *lead to death of 7/7 embryos in 5 days (Panel A), whereas infection with 4,500 CFU of the Δ*iglA *strain (ODB2) lead to the death of 1/7 embryos in 6 days (Panel B).

When the Δ*iglA *strain was used to infect chicken embryos it caused low mortality when compared to wild type *F. novicida *(Fig. 10). The wild type strain of *F. novicida *caused 100% mortality at day 5 post infection at an infecting dose of 600 CFU, whereas the Δ*iglA *strain caused only 14% mortality at day 6 with an infecting dose of 4,500 CFU (Fig. 10) or 50% mortality at day 6 with an infecting dose of 45,000 CFU (data not shown).

## Discussion

There is growing evidence that the *iglABCD *operon is needed for *F. tularensis *intracellular growth and virulence and that the MglAB proteins are involved in regulating the expression of *iglABCD*. However, there is very little genetic and corresponding biochemical data demonstrating the roles of MglAB and IglAB and their corresponding homologues in other bacteria. For example, while it is clear that MglA plays a role in regulating the amount of *iglABCD *transcript it is unclear if the role precisely corresponds to that of the *E. coli *SspA protein. The data that exists for the functioning of SspA suggest that much of the regulation of stationery phase proteins occurs indirectly via the repression of H-NS, and that some of the effect of SspA is post-transcriptional [14].

There is also growing evidence that proteins encoded by IAHP clusters, of which IglAB homologues are important components, are involved in secretion of proteins from gram-negative bacteria [24,27]. There are approximately 30 homologues of *iglAB *and in every case the two genes are adjacent to each other and arranged in the same gene order. In this work we provided biochemical evidence that the IglAB proteins physically associate with each other and are localized to the cytoplasm. The surprising finding that inactivation of the *iglB *gene results in the disappearance of the IglA protein suggest that the presence of IglB is required for IglA to be stable.

IglA was first identified as a locus that when inactivated by a transposon insertion rendered *F. novicida *defective for growth in macrophages [17]. However, it could not be ruled out that the effect was due to interruption of transcription of downstream genes. In this report, we provide strong evidence that IglA is necessary for intracellular growth as a non-polar *iglA *deletion mutant was defective for growth in a mouse macrophage-like cell line. *In cis *complementation of the Δ*iglA *strain restored intramacrophage growth although the growth was slower than in the wild type strain. The *in cis *complementation strategy created two *iglA *promoter regions on the chromosome, one on either side of a kanamycin resistance cassette. It is conceivable that this results in aberrant regulation of *iglA *expression, which could explain why the growth of the complementation strain lags early during infection. We were unable to complement the *iglA *deletion mutant *in trans *with pFNLTP1::*iglA*, a high copy-derivative of an endogenous *Francisella *plasmid. Presumably, over-expression of IglA was lethal to *F. novicida*.

We hypothesize that IglA and IglB are cytoplasmic, chaperone-like proteins that are involved in secretion of virulence factors. Therefore, the biological significance of IglAB interaction may be to secrete *Francisella *effector molecules. In other pathogens, secretion of virulence proteins often requires interaction between two cytoplasmic proteins. For example, in *Yersinia pestis*, a complex composed of SycN and YscB function as chaperones for YopN [28], which is secreted to the cell surface [29]. Also, interaction of IcmS and IcmW is required for translocation of effector proteins via the Dot/Icm complex during *Legionella pneumophila *intracellular growth [30,31]. Hager *et al. *recently demonstrated protein secretion by *F. novicida *[32]. We did not observe any difference in secreted peptides between broth-grown wild type *F. novicida *and the Δ*iglA *strain by SDS-PAGE electrophoresis (data not shown). This observation is not surprising given the fact it has been demonstrated that secretion involving IAHPs is a highly regulated or an *in vivo-*induced process [27].

In summary, our results suggest that IglA and IglB are interacting cytoplasmic proteins that are required for intramacrophage growth. The significance of the interaction may be to secrete effector molecules that affect host cell processes.

## Conclusion

The *Francisella *Pathogenicity Island harbors uncharacterized genes implicated in virulence. By constructing an in-frame deletion mutant we have shown that the FPI gene *iglA *is needed for intramacrophage growth. Biochemical characterization of IglA strongly suggests that it is a cytoplasmic protein that interacts physically with IglB. In addition, we provide data that show IglA is induced during infection of macrophages. Bioinformatics analysis of the FPI suggests that it is similar to virulence loci that encode a protein secretion apparatus. We propose that IglA and IglB are chaperone-like proteins that are part of a secretion system in *F*. *novicida*.

## Methods

### Bacterial strains and culture conditions

All strains used in this work are listed in Table 1. *F. novicida *strains were grown in trypticase soy broth supplemented with 0.1% cysteine (TSBC) or on trypticase soy agar supplemented with 0.1% cysteine (TSAC) unless stated otherwise. Kanamycin (45 μg/ml) or erythromycin (30 μg/ml) or 10% sucrose were added as needed.

### Subcellular fractionation

1000 ml of overnight *F. novicida *U112 culture was harvested and resuspended in 50 ml of cold phosphate buffered saline (PBS). Cells were broken by repeated passage through a French Pressure cell (American Instruments Co, Silver Spring, MD) at 1200 PSI. Unbroken cells were removed by 20 min of centrifugation at 10,000 × *g *at 4°C, and a sample was taken as the total protein fraction. The lysate was subjected to ultracentrifugation (Beckman L8-70, rotor Type 45 Ti) for 1 hr at 100,000 × *g *at 4°C to pellet the membranes. The supernatant (soluble protein fraction) was removed, whereas the membrane pellet was resuspended in 2.5 ml of 1% Sarkosyl (Sigma). The sarkosyl soluble (inner membrane) and the sarkosyl insoluble (outer membrane) were separated by ultracentrifugation for 1 hr at 100,000 × *g *at 4°C in a Beckman TLA-100.3 ultramicrocentrifuge. The activity of the inner membrane-associated enzyme NADH oxidase was determined per mg of protein [33] for each of the fractions as a measure of the relative mixing of the different cell compartments. The soluble fraction contained 3%, the sarkosyl soluble membrane fraction 79% and the sarkosyl insoluble membrane fraction 18% of the NADH oxidase activity. In addition, we found that 90% of IglC was found in the soluble fraction (data not shown) and 10% was in the total membrane fraction. IglC could not be detected in the sarkosyl-soluble or sarkosyl-insoluble membrane fractions. As IglC has previously been shown to be a soluble protein [16], this served as another control of our fractionation experiment. Isolation of periplasmic proteins was performed as described by Nossal and Heppel [34].

### Co-immunoprecipitation

500 μl of soluble fraction was pre-cleared by incubation with 20 μl protein-G/Agarose beads (40% slurry; EMB Bioscience, La Jolla, CA) and 10 μg nonspecific antibody for 1 h at room temperature (RT). Beads and bound proteins were removed by centrifugation and the soluble fraction was incubated with 10 μl rabbit anti-IglA serum or nonspecific antibody for 1 h at RT followed by addition of 75 μl protein-G/Agarose beads and incubation 1 h at RT. Complexes were recovered by centrifugation, 6500 rpm, 3 min, and beads were washed three times with 150 mM NaCl, 10 mM Na_2_H_3_PO, pH 7.2. After the final wash, complexes were resuspended in 30 μl SDS-PAGE loading buffer and the sample was boiled for 5 min. Beads were removed by centrifugation and released proteins were separated on a 12% Sodium dodecyl sulphate-polyacrlamide electrophoresis (SDS-PAGE) gel. The immunoprecipitated material was examined by immunoblotting with anti-IglA to confirm that IglA was present (data not shown).

### SDS-PAGE and Western blotting

To normalize the amount of protein added to each lane, the concentration of protein samples were determined by use of the BCA assay (Pierce). SDS-PAGE was performed according to standard techniques. Separated proteins were transferred onto a Trans Blot^® ^nitrocellulose (BioRad) or Immobilon-FL (Millipore) membrane and blocked with 5% skim milk (Difco) in PBS. Anti-IglA, and anti-IglC antibody were used at dilutions of 1:4,000 and 1:500 respectively. To detect bound antibody blots were incubated with IRDye800DX-conjugated goat anti-rabbit or IRDye700DX-conjugated goat anti-rat immunoglobulin G (Rockland, Gilbertsville, Pa.) and visualized in a LiCor Odyssey imaging system.

### MALDI-TOF

Following SDS-PAGE separation of proteins in-gel digestion with trypsin was carried out, and peptides extracted. 10 μl of the peptide sample was loaded on to a C18 zip tip and washed three times in 10 μl of 0.1% TFA and eluted with 2 μl of 50% ACN and 0.1% TFA containing 10 mg/ml 4-hydroxy alpha cyanocinnamic acid. MALDI-TOF MS analysis of the peptides was carried out using a Voyager-DE STR (Applied Biosystems, Foster City, CA). Mass fingerprint analysis was done using Mascot (Matrix Science, UK).

### Construction of *iglA *deletion mutant

IglA deletion mutant, ODB2, was constructed using a two-step integration-excision method. 1.5 kilobasepair (kbp) regions flanking *iglA *were amplified with primers iglA L-F 5' cgcggccgcagcaaaaatgctggaggtgt, iglA L-R 5' cctcgagcatcaaccttgaatttgggatt, for the left-hand flanking region, and with primers iglA R-F 5' cctcgagctcttgtgatgctgctgagtct, iglA R-R 5' cgcggccgcaataccagccaggcttaccc, for the right-hand flanking regions. These were cloned into plasmid pCR2.1 (Invitrogen) and verified by sequencing. The flanking regions were then joined by ligation. The flanking region construct was ligated to an erythromycin resistance-*sacB *cassette and the ligation mixture was used to chemically transform *F. novicida *JL0 to erythromycin resistance as previously described [35]. The JL0 strain (Ludu et al., unpublished data) is a derivative of the *F. novicida *U112 prototype strain that has a deletion in a sucrose hydrolase gene, and thus is sensitive to *sacB *expression in the presence of sucrose. An erythromycin resistant colony was grown and plated on TSAC containing 10% sucrose which acts as a counter selective marker for the *sacB *gene. Sucrose sensitive strains were examined for loss of *iglA *by PCR.

The *iglA *and *iglB *allelic replacement mutants, ODB7 and ODB1, were constructed as previously described [12]. Briefly, 1.5 kbp regions flanking *iglB *were PCR amplified with primers iglB L-F 5' cgcggccgcgaagaagataattcttcttctgaaaccg, iglB L-R 5' cctcgag attgtcataacaaaatcctctctactt, iglB R-F 5' cctcgagtgactatagatactaggcttgaacca, iglB R-R 5' cgcggccgctcaaaggcttttggaaatcaa incorporating Xho I sites and ligated to an erythromycin resistance cassette with added Xho I sites. *F. novicida *U112 was transformed with the construct as previously described [35]. The same primers used for construction of ODB2 were used for ODB7.

### *In cis *complementation

IglA and its promoter region were amplified with primers IglA int-L 5' CCCCTCGAGAGCCGTTTTCAATATTGGTTT and IglA int-R 5' CCCCTCGAGCAACTTCTGTAGATCCCCCAAA incoporating added XhoI sites and ligated to a kanamycin resistance cassette carrying a *F. novicida *promoter (Ludu et al., unpublished data). The construct was used to transform ODB2 as previously described [35].

### Macrophage infection assay

Macrophage infection assays were performed essentially as described previously [2]. Briefly, J774.1 mouse macrophage-like cells were infected with *F. novicida *strains at a multiplicity of infection of 50:1 (bacterium-to-macrophage), and monolayers were incubated for 2 h in Dulbecco's Modified Eagle Medium containing 10% fetal bovine serum (DMEM), washed five times in Dulbecco's Phosphate Buffered Saline (DPBS), and incubated at 37°C in 5% CO_2_. Macrophages were lysed in 0.1% deoxycholate at 0, 24, 48 and 72 h post infection. To determine bacterial growth, lysed macrophages and culture supernatants were serially diluted in DPBS and plated on TSAC. As *F. novicida *does not grow in DMEM, this allows for an adequate determination of intracellular growth [2].

### Chicken embryo infections

Fertilized White Leghorn eggs were obtained from the University of Alberta Poultry Research Station. Seven-day old embryos were injected under the chorioallantoic membrane with various doses of 100 μl of *F. novicida *diluted in PBS as previously described [36]. The embryos were monitored for death for 6 days.

## Authors' contributions

OMB performed all experiments, constructed *iglA *and *iglB *mutants and drafted the manuscript. JSL constructed the deletion of the sucrose hydrolase gene in *F. novicida*. FEN was the principal investigator and supervised the project. All authors read and approved the final manuscript.

## Figures and Tables

**Table 1:** Strains and plasmids used in study.

Name	Phenotype/Relevant Characteristics	Reference or Source
U112	*Francisella novicida *prototype strain.	ATCC
JL0	U112, Δsucrose hydrolase strain used to make deletion mutants.	Laboratory strain
ODB2	JL0, Δ*iglA*	This study
ODB7	U112, *iglA*::Em^R^	This study
ODB1	U112,*iglB*::Em^R^	This study
ODB5	Δ*iglA*/*iglA*:Km^R^, *in cis *complementation of *iglA *in strain ODB2	This study
CG62	U112, *iglC*::Tn*Max*2	Gray *et al. *(2002)
GB2	U112, *mglA*	Baron *et al. *(1998)
GB6	U112, *mglB*::mTn *10*Km	Baron *et al. *(1998)
DH5α	F^- ^Φ80d*lac*ZΔM15 Δ(*lac*ZYA-*arg*F)U169 *rec*A1 *end*A1 *hsd*R17(r_k _^-^, m_k _^+^) *pho*A *sup*E44 *thi*-1 *gyr *A96 *rel*A1 λ^-^	Invitrogen
pCR2.1	Cloning vector, Amp^R ^Km^R^	Invitrogen
